# Storage Properties of Double-Layer Films Enriched with *Phytolacca americana* L. Extract as Active Packaging for African Catfish, with a New Approach to Antioxidant Film Assay and Additional Analysis of *P. americana* Extract Toxicity on Human Cell Lines

**DOI:** 10.3390/molecules30071447

**Published:** 2025-03-25

**Authors:** Joanna Maria Jasińska, Klaudia Michalska, Joanna Tkaczewska, Katarzyna Tkacz, Arkadiusz Zakrzewski, Agnieszka Galanty, Iwona Kamińska, Maria J. Chmiel, Ewelina Jamróz

**Affiliations:** 1 Department of Chemistry, University of Agriculture, ul. Balicka 122, 30-149 Kraków, Poland; ewelina.jamroz@urk.edu.pl; 2 Department of Phytochemistry, Maj Institute of Pharmacology, Polish Academy of Sciences, ul. Smętna 12, 31-343 Kraków, Poland; klaudiaz@if-pan.krakow.pl; 3 Department of Animal Product Technology, Faculty of Food Technology, University of Agriculture, ul. Balicka 122, 30-149 Kraków, Poland; joanna.tkaczewska@urk.edu.pl; 4 Department of Food Microbiology, Meat Technology and Chemistry, Faculty of Food Sciences, University of Warmia and Mazury, Plac Cieszyński 1, 10-719 Olsztyn, Poland; ktkacz@uwm.edu.pl (K.T.); arkadiusz.zakrzewski@uwm.edu.pl (A.Z.); 5 Department of Pharmacognosy, Jagiellonian University, Medical College, ul. Medyczna 9, 30-688 Kraków, Poland; agnieszka.galanty@uj.edu.pl; 6 Department of Botany, Physiology and Plant Protection, Faculty of Biotechnology and Horticulture, University of Agriculture in Krakow, al. 29 Listopada 54, 31-425 Kraków, Poland; iwona.kaminska@urk.edu.pl; 7 Department of Microbiology and Biomonitoring, Faculty of Agriculture and Economics, University of Agriculture in Krakow, al. Mickiewicza 21, 31-120 Kraków, Poland; maria.chmiel@urk.edu.pl; 8 Department of Product Packaging, Cracow University of Economics, ul. Rakowicka 27, 31-510 Kraków, Poland

**Keywords:** films, packaging, biodegradable, bioplastic, furcellaran, *Phytolacca americana* extract, double layer active films, African catfish, human cell lines

## Abstract

Novel double-layer films based on furcellaran (FUR) and gelatin (GEL) with the addition of *Phytolacca americana* L. (PA) extract were used as active packaging for African catfish fillets. Films with PA extract have been shown to minimize the catfish spoilage effects, expressed as odor reduction compared to control samples; however, neither the films nor the PA extract exhibited antimicrobial activity against tested groups of microorganisms (fungi, lactic acid bacteria, *Enterobacterales* and psychrotrops) or specified microorganisms (*E. coli*, *S. aureus*, *S. cerevisiae*). The tested films demonstrated antioxidant activity determined by the DPPH, ABTS, FRAP, CUPRAC and Folin–Ciocâlteu methods. Cytotoxicity analysis showed that the PA extract affected tested cell lines (PNT2—prostate epithelial cells, HepG2—human liver cells, HaCaT—normal human keratinocytes and Nty-hori 3-1) only to a small extent—the calculated IC_50_ values exceeded the maximal tested concentration of 500 µg/mL.

## 1. Introduction

Food packaging is a key part of the food industry because it ensures the quality and safety of products during transport while maintaining stable storage [1]. Packaging based on frequently used petroleum-based materials (i.e., polyethylene, polystyrene) pose a global environmental problem by generating large volumes of accumulating waste (these materials are non-biodegradable) [2,3]. Modern plastic waste management systems rely on landfilling, incineration, composting and recycling [4]. However, the incineration process of synthetic plastics causes the release of harmful gases (i.e., NO, CO, SO_2_ or HF) into the environment [5,6]. The chemicals being used in the production of petroleum-based synthetic packaging materials can have negative impacts on the human body (by causing hormonal changes and neurological damage and increasing cancer morbidity) [7]. These plastic materials enter the aquatic ecosystem and break down into microplastics and are transported into the food chain via marine and wild life [7]. As a result of growing public awareness, biodegradable alternatives are being sought, such as films based on natural polymers, which have great potential to replace non-biodegradable materials [8,9,10].

Films based on compounds of natural origin are already being examined as plastic substitutes. According to Srinivasa et al. [11], mango fruits (*Mangifera indica*) stored in carton boxes, the top surfaces of which were covered with chitosan films, demonstrated an extension of shelf life by up to 6 days without any microbial growth or off-flavor in comparison to mangos placed in boxes covered with low-density polyethylene (LDPE) films. Moreover, films based on natural polymers are often enriched with plant extracts, which are responsible for the active and intelligent aspects of packaging. According to Romero, Cruz, Díez-Méndez and Albertos [1], pectin-based films with blueberry extract protected the salmon samples from deterioration due to their anthocyanin content and antioxidant capacity, increasing the shelf life of salmon.

*Phytolacca americana* L. (pokeweed) is a native plant of North and South America; however, it is now spread worldwide and can even be found in Central Europe [12,13]. In its native occurrence, the plant parts were used as traditional medicine in treating disease through antimicrobial, anti-inflammatory and anticancer activity [14]. The berries of *P. americana* are rich in phenolic acids and flavonoids and contain betalains (betacyanins and betaxanthins). Nonetheless, due to the possible presence of toxic compounds in this plant (mitogen, saponins), it is currently considered toxic [15,16,17].

African catfish (*Clarias gariepinus*) belongs to the *Clarias* genus, *Clariidae* family, Siluriformes order (catfish) [18]. This fish naturally occurs in the areas of Central and South Africa and the Middle East [19]. African catfish is a high-protein food source that is popular among consumers for the taste of the meat and is perfect for processing as well as soup production [20]. This species is being cultured; however, the catfish meat is highly perishable. Thus, novel methods of packaging and storage need to be explored [21,22].

In the previous work [23], the full functional characteristics with ecotoxicity and biodegradability of the double-layer films are presented, while the aim of the following study was to use double-layer films (based on furcellaran and gelatin) with the addition of an active ingredient (*P. americana* extract) as a packaging material for catfish. This kind of packaging can be an alternative that can replace the standardly used plastic-based films in the food sector. An additional aim of the work was to explore the new approach to determine the antioxidant activity of the films. Furthermore, in this research, the potential toxic effect of the *P. americana* berry extract was determined against chosen human cell lines (human prostate epithelial cells (PNT2), thyroid follicular epithelial cells (Nthy-ori 3-1), hepatocellular carcinoma (HepG2) and skin keratinocytes (HaCaT)) to ensure the safe future usage of films enriched with *P. americana* extract in the food industry.

## 2. Results and Discussion

### 2.1. The Impact of Phytolacca americana Extract on Cell Cultures

The safety of the extract from the fresh fruit of PA was tested using a panel of normal, non-neoplastic cells, representing different organs, namely the prostate, thyroid and skin. Moreover, to verify the hepatotoxic potential of the extract, hepatocellular carcinoma HepG2 cells were also included in the study. Despite its cancer origin, this cell line is used as a standard in hepatotoxicity studies, as HepG2 cells reveal similar phenotypic characteristics and functional properties to normal hepatocytes [24].

The obtained results clearly indicate that the examined extract affected the viability of the cells used in the experiment, but only to a small extent (Table 1). The effect was slightly stronger after longer exposure of the cells to the tested extract; however, the calculated IC_50_ values exceeded the maximal tested concentration of 500 µg/mL. According to the criteria of the National Cancer Institute and the Geran protocol [25], extracts with an activity of IC_50_ > 500 µg/mL are classified as non-cytotoxic.

### 2.2. Antimicrobial Properties of Films and Phytolacca americana Extracts

The tested films showed unusual properties against the tested microorganisms (the films showed an effect resembling the so called “eagle effect”) or no antimicrobial activity at all (according to both A1 and A2 methodologies). Additionally, no antimicrobial effect was presented by PA extracts. According to the literature, extracts from PA berries demonstrate strong inhibitory activity against *E. coli*, *M. furfur*, *S. epidermidis*, *V. parahaemolyticus* and *L. monocytogenes* [26]. Because of high dilution and the appearance of polymers in the film matrix (which constitute a good medium for microbes), the inhibitory effect did not appear. In the case of *S. aureus* ATCC 6538, an “eagle-like effect” occurred in all of the tested films [27]. This is a phenomenon in which bacteria or fungi exposed to antibiotics at concentrations higher than the optimal bactericidal concentration (OBC) have a paradoxically higher rate of survival than those exposed to OBC, due to a reduced rate of net cell death. Despite extensive research on this effect, its mode of action is not well understood. Although the “eagle effect” resembles antibiotic persistence, there is strong evidence that these phenomena are substantially different phenotypic responses to antibiotic treatment [27]. This effect is known in in vitro research and occurs mostly for antibiotics [28]. However, in this study, it was present in all the film samples (including the control) and, what is more important, without any added antibiotics. The results obtained by Jamróz, Kulawik, Krzyściak, Talaga-Ćwiertnia and Juszczak [29] showed that FUR/GEL films do not have antimicrobial activity; however, when films were enriched with tea extracts (pu-erh or green tea), the antimicrobial activity was visible against *E. coli* 26,922 and *S. aureus* ATC25923, but no activity against *Candida albicans* ATCC 90,028 or *Hanseniaspora uvarum* was noticeable. Due to the fact that there was no resemblance to the “eagle effect”, but the *S. aureus* strains were different, there is a possibility that it was a specific reaction of this strain (presumably, that the furcellaran-gelatin complex somehow reacted with the bacterial ability to decompose this specific linking). In addition, there is a possibility of polyphenol-protein complex formation that could increase the antimicrobial effect of phenols (of which PA berries are rich); however, the control sample did not contain the PA extract [30,31,32].

Additionally, the results from analyses of PA extracts did not indicate any antimicrobial activity (this may have been due to their high dilution). Boo, Park, Woo and Park [26] presented the antimicrobial activity of a PA berry extract against *Escherichia coli*, *Malassezia furfur*, *Staphylococcus epidermidis, Vibrio parahaemolyticus* and *Listeria*, although the tested extract was more concentrated (100 mg/mL). Hamissou and Kurdmongkoltham [33] reported that the extract from PA berries had had a negative effect on the log phase of the growth curve of *E. coli*; however, the initial pokeweed berry extract had a concentration of 250 mg/mL. According to DemİRkan et al. [34], the pokeweed fruit exocarp extract at concentrations of 10/20/40 mg/mL, respectively, did not show any inhibitory activity against *E. coli*, *E. faecalis*, *S. typhimurium*, *S. aureus*, *Y. entercocolitica* or *K. pneumoniae*. Nonetheless, at a concentration of 60 mg/mL, an inhibitory effect was noticed against *K. pneumoniae*, and at a concentration of 80 mg/mL, such an effect was also reported against all the tested microbes, apart from *Y. entercocolitica*. Additionally, an inhibitory effect was noticed for the pokeweed berry pulp extract (only for 60 mg/mL and 80 mg/mL concentrations) against *S. typhimurium* and *K. pneumoniae*. Furthermore, the seed extract at concentrations of 10/20/40/60/80 mg/mL was tested, with only the 10 mg/mL extract not showing inhibitory activity against *K. pneumoniae*.

### 2.3. Antioxidant Properties of Films

The antioxidant properties of films are responsible for active aspects of natural packaging by prolonging the shelf life of food by retarding the rate of oxidation reactions. As of today, only a small part of the created films have been successfully taken from the laboratory to commercial usage [35]. Fruit extracts are currently considered to be a valuable addition to the polymer film matrix due to their high content of antioxidants, which can also promote better UV barrier properties of films. The addition of the PA extract ensures better protection against UV radiation, which can contribute to prolonged food storage [23]. The fruits that contain anthocyanins (colors of which are purple, blue and red) show the highest total antioxidant capacity [36]. However, not all fruit color range is determined by anthocyanins—the color of *P. americana* berries results mostly from betalains—color range from red-violet (betacyanins) to yellow (betaxanthins) [37]. For most families of the Caryophyllales species (including *P. americana*), the anthocyanins in the fruits are being replaced by betalains [38]. According to Jerz et al. [39], there are various betalains in the *P. americana* berry extract (Betanin, Isobetanin, 2′-Apiosylbetanin, 6′-O-Salicylbetanin and 5″-O-Salicyl-2′-O-apiosylbetanin). The *P. americana* berries are also enriched with phenolic acids—Marinas et al. reported that, the main phenolic acids quantified in the alcoholic extract of P. americana fruits were cinnamic acid (1019.98 μg/L), chlorogenic acid (283.65 μg/L) and vanillic (104.28 μg/L), syringic (99.31 μg/L) and ellagic acids (73.99 μg/L) [40]. In our previous study, the selected compounds of the *P. americana* extracts used for film production were evaluated, and the results confirmed the high content of betacyanin and betaxanthin (9.15 μg/mL and 6.84 μg/mL for the most concentrated extract, respectively). The phenylpropanoid content ranged from 12.40 μg/mL to 71.40 μg/mL, and the total phenolic content was within the range of 63.13 μg/mL and 486.11 μg/mL [23]. In the present study, the antioxidant properties of films have been tested using multiple methods. The results of those assays are presented in Table 2.

#### 2.3.1. Iron Ion Reduction Ability

Due to the different conditions of the FRAP and CUPRAC reaction methods, the use of both of them allows for more exact estimation of the ion reduction power activity. The FRAP assay is carried out at pH = 3.6, which is much lower than the physiological pH and is insufficiently responsive to thiol- type (i.e., -SH containing) [41,42]. Moreover, the antioxidants detected by FRAP are limited to water-soluble ones (i.e., soluble in aqueous ethanol solutions); thus, carotenoids have no ferric-reducing ability [43]. Additionally, the CUPRAC redox reaction is carried out at a nearly physiological pH level (pH 7 of ammonium acetate buffer) in comparison to the unrealistic acidic conditions (pH 3.6) of FRAP or the basic conditions (pH 10) of the Folin–Ciocalteu assay. However, in more acidic conditions than the physiological pH, the cupric-reducing capacity may be suppressed due to protonation on phenolics, whereas in more basic conditions, proton dissociation of phenolics (converted into phenolates) would enhance a sample’s reducing capacity [44]. For the FRAP method, no antioxidant power was presented by the control film. Addition of the PA extract (at concentrations of 4% and 6%) resulted in efficient incorporation of reducing agents into the films, significantly enhancing their antioxidant power up to 11 mM of Trolox equivalents per 1 g of film (Table 2). In comparison, the results obtained in our previous research with the FRAP assay (where the same films were tested, but with different methodology—liquid extracts) were lower (the highest value was 3.40 mM TE·g^−1^ for 6% enriched films). Nevertheless, the values also increased with a higher addition of the PA extract [23]. The results of the CUPRAC assay showed significant differences between the control (approx. 20 mM TExg^−1^) and enriched films (approx. 4 mM TExg^−1^ 6% PA addition), with the highest result obtained for the control. These results are different from those obtained in our previous research, in which all of the achieved values were at the level of approximately 50 mM TE·g^−1^. These differences were dependent on extract preparation (in our first study, a standard film liquified procedure was used, while in this one, solid film samples were implemented), and the higher value for the control samples may be due to the formation of polyphenol-protein complexes in water once released from the matrix film, which may be deleterious to the antioxidant activity [30]. The interaction of polyphenols (from the PA extract) with proteins (gelatin) can negatively affect the antioxidant efficacy of these compounds. The degree of masking the antioxidant activity by these complexes depends on the polyphenol composition and type of protein [45,46]. The formation of these polyphenol-protein complexes occurs as a result of many weak interactions (mainly hydrophobic) and is dependent on temperature, pH, the nature and concentration of the protein and polyphenol, etc. [46,47]. However, these formed polyphenol-protein complexes may retain part of the antioxidant activity if some hydroxyl groups remain free to allow the molecule to act as an antioxidant [48].

#### 2.3.2. Free Radical Scavenging

The results from DPPH assay indicated no significant differences between films (approx. 6.5%) (Table 2). However, the results were higher than those obtained with the liquid extract methodology (approximately 2%) [23]. Additionally, the DPPH activity of the PA extracts was up to 10% for the highest concentration (2.16 mg/mL). These differences between results from various film testing methodologies and PA extracts may be due to the effect of the differing pH of the suspension in which the reaction is carried out—for the liquid extract there is a possibility of a higher amount of hydrogen ions (coming from gelatin, which was previously exposed to a high temperature in high humidity). According to Dawidowicz et al. [49], high hydrogen ion concentrations can affect the antioxidant properties of compounds measured by the DPPH method; thus, it can be assumed that the increase in hydrogen ion concentration slows down DPPH/antioxidant reaction kinetics. However, according to Kanatt [50], films based on polyvinyl alcohol and gelatin with the addition of an *Amaranthus* leaf extract (ALE) (which belongs to the same order as pokeweed and also contains betalains) showed a DPPH activity at the level 42%, and the ALE DPPH activity was estimated as 80% at a concentration of 1 mg/µL). The PA extracts increased the ABTS radical cation scavenging potential of the tested films from 30.43% (control films) to 72.75% (4% PA enriched films) (Table 2). Similar results were reported by Hanani, et al. [51] for films based on fish gelatine supplemented with polyphenol-rich pomegranate peel powder (PPP). They demonstrated activity for the control films at the level of 53%, and for films with 5% PPP, at the level of 71.82%. According to our previous study [23], the results for the ABTS assay obtained with a liquid film extraction were lower—the activity was at the level of approx. 20%, and in this study (with solid samples), the activity was at the level of approx. 70%. Additionally, the extracts of PA were characterized by an ABTS scavenging activity at the level of approx. 45% for the highest concentration (2.16 mg/mL). The results obtained in this assay indicate a possible scavenging activity of the polymer matrix (which was also noted in our previous research) but also the possibility of a polymer matrix and extract combination reinforcing effect on antioxidant activity. These differences between the obtained values for two methodological assays conducted on the same samples may be due to the potential formation of a polyphenol-protein complex [30]. In addition, the temperature and pH could have affected the results. According to Otálora González et al. [52], betalains (betaxanthins, betacyanins), with which the PA extract is enriched, are more stable in lower temperatures (0–25°) [39]. Khan and Giridhar [53] stated that the degradation of betalains accelerates with increasing temperature and heating period. This is especially important information, because in our previous research, we used a methodology with the film extract heating procedure, and the values from those performed assays were lower [23].

#### 2.3.3. Total Reducing Power

The total reducing power (TRP) of the tested films varied from 5 to 36 mg CAExg^−1^ and was positively correlated with PA extract concentrations; however, pure PA extracts demonstrated very low TRP values (<1.0 mg CAExg^−1^) [23]. In addition, in our previous study, the TRP of films was lower (approx. 21 mg CAExg^−1^). According to Antony and Farid [54], for commonly performed extractions (such as solvent, Soxhlet, ultrasound-assisted and subcritical water extractions), the TPC is highest at an extraction temperature between 60 and 80 °C, but compared to the results of our preview article [23] and the present research, the highest TPC was presented by films incubated directly into the extraction solution at room temperature.

### 2.4. African Catfish Quality During Storage

#### 2.4.1. pH Value and Color Parameters of African Catfish Fillets

The pH rate of fish is an indicator reflecting the freshness of fish quality. A change in the pH of catfish meat during cold storage is illustrated in Table 3. It can be seen that the pH of all groups demonstrated a comparable decreasing and then increasing trend. During the early stage of chilling, the pH values were decreased, which could have been due to the glycolysis of catfish fillets that led to the accumulation of lactic acid and the ATP to release inorganic phosphate. With the extension of refrigeration time, the autolysis of the fish protein and the formation of trim-ethylamine as well as ammonia caused by the action of microorganisms has been shown to lead to a gradual increase in pH value [55]. During the storage period, compared to the control group, the pH values of both CA and A increased more slowly. However, no statistical differences were observed between the pH of the samples tested. The color of food is important in consumer assessment of its quality. Therefore, the effect of active packaging on the color of samples is a significant quality parameter. The results for surface color (L*, a* and b* values) of the catfish fillets covered with the film during storage are shown in Table 3. In general, with the application of the films, the brightness (L* value) and yellowness (b) decreased, whereas the redness (a* value) of the catfish fillets increased compared to the control samples (without covering). Jung, et al. [56] stated that color changes in fish meat during storage can be affected by both enzymatic and non-enzymatic processes, resulting in myofibrillar protein breakdown and myofibril disorganization. Furthermore, Zeng et al. [57], claimed that a Maillard reaction between the fish protein and carbohydrates from the film may have occurred, whereas in acidic conditions, yellow pigments may have been created, leading to the degree of color change being affected by processing conditions and moisture content. It is worth noting that the inclusion of plant extracts in films offers an appropriate light barrier, which is also necessary for preventing oxidation processes [58].

#### 2.4.2. Sensory Evaluation of African Catfish Fillets

Sensory characteristics, including smell, color and surface of the meat, and overall score for the fish fillets under control conditions and treated with packaging were compared. The sensory evaluation showed a significant decrease in acceptance for all samples during the course of the storage duration (Table 4). The panelists noticed an increase in unpleasant off-odor during the storage period, especially in the control sample starting on days 5 and 6 of storage (scores of 2.6 and 1.38, respectively). In the other test groups, the panelists observed lower odor intensity on subsequent storage days. Compared with the control treatment, the intensity of the purple color in the sample-covered film with the pokeweed addition was greater. The purple color of A samples comes from the pokeweed pigments called betacyanin that are present in the films, which migrate from the film to the product when exposed to water contained in the fish. Betacyanin has nitrogen-containing pigments that are water-soluble and that were probably not properly bonded by the film components [50].

The sensory evaluation results are usually correlated with microbial and chemical value analyses [59]. Due to high lipid oxidation and microbial growth, both the control samples and study groups of catfish fillets showed spoilage, appearing as an off-odor after four days of storage. The preservative effects of the coating with pokeweed addition have been shown to minimize the spoilage effects, expressed as odor reduction (the scores of odor for sample A were 4.50, 2.86 and 3.10 on the following days of storage vs. the score for control samples: 4.28, 2.60 and 1.38). Other authors [60] have concluded that fish treated with biodegradable films and natural preservatives present better or similar sensory characteristics compared to the control treatment, which is supported by our results. 

#### 2.4.3. Microbial Quality of African Catfish Fillets

**Figure 1:**
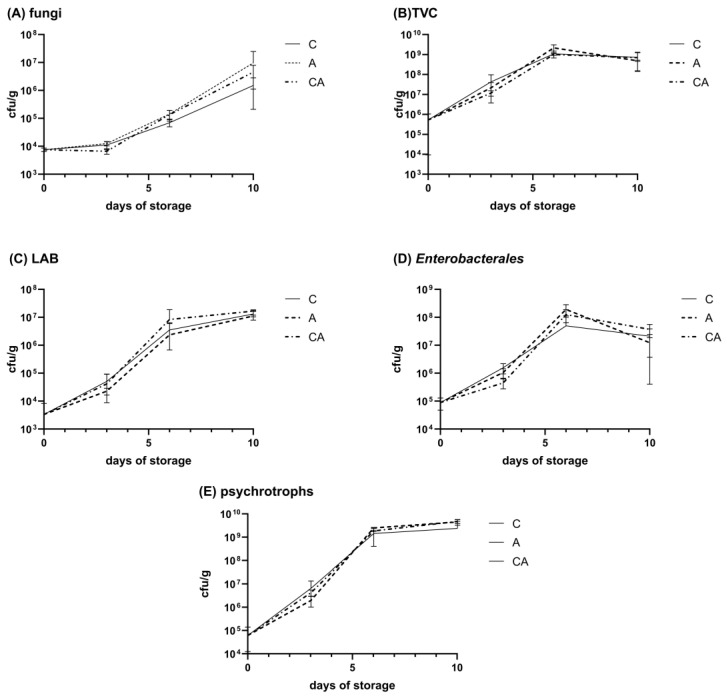
Microorganism growth during storage of catfish; C—control; CA—film without pokeweed; A—film with added pokeweed; (**A**)—fungi, (**B**)—total viable counts—TVC, (**C**)—lactic acid bacteria—LAB, (**D**)—*Enterobacterales*, (**E**)—psychrotrophs.

During storage, it was not observed that the films possessed any bactericidal or bacteriostatic properties (Figure 1).

In the case of fungi, the most significant differences were noted between the application of the films, with counts of 4.49 × 10^6^ cfu/g for the film without the addition of PA and 9.66 × 10^6^ cfu/g for that with the inclusion of PA, compared to the control sample at 1.51 × 10^6^ cfu/g. However, these results were not statistically significant (*p* > 0.05). The most pronounced growth was observed for psychrotrophs, exhibiting an increase of up to five orders of magnitude for both the control sample and samples with films. A substantial growth of four orders of magnitude was observed for LAB. For the remaining groups, an increase of three orders of magnitude was recorded. In analyzing the growth of the *Enterobacterales* order, a slight decline in the number of viable cells was noted between days 6 and 10, though these differences were not statistically significant (*p* > 0.05).

Özvural et al. [61] claimed that the encapsulation and binding of plant extracts or other active components in the coating may prevent its antimicrobial activity. Additionally, the release rates and behavior of antimicrobial substances depend on several factors, including polymer types, the method and process of film preparation, film microstructures, antimicrobial-polymer interactions and environmental as well as medium conditions. There are many factors affecting the rate of microbial spoilage of fish [62]; therefore, further research should be conducted to clarify the interactions of the tested coatings with the model fish products.

## 3. Materials and Methods

### 3.1. Films

**Figure 2:**
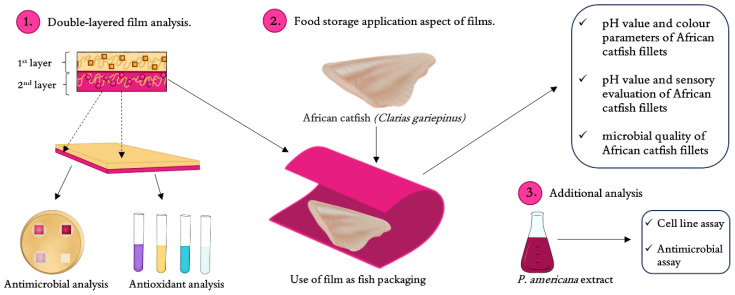
Diagram showing activities and analyses performed to assess packaging potential of double-layer films enriched with pokeweed extract.

The double-layer films were prepared according to the methodology presented by Jasińska, Michalska, Szuwarzyński, Mazur, Cholewa-Wójcik, Kopeć, Juszczak, Kamińska, Nowak and Jamróz [23]. Briefly, the *Phytolacca americana* (PA) ripe berries (of which seeds were obtained from the Botanical Garden of the College of Nyíreghyáza (Hungary) and were grown in the Garden of Medicine Plants of the Maj Institute of Pharmacology, Polish Academy of Sciences, Kraków, Poland) were extracted with methanol, which was then evaporated. Next, 3.5 g of the crude extract was diluted with 96.5 g of distilled water and used as a stock solution, which was then added to the first film layer at different concentrations. The films consisted of two layers: the first based on furcellaran (Est-Agar AS, Estonia) and gelatin (Sigma-Aldrich, Poznań, Poland) and the second based on furcellaran with the addition of the PA extract (concentrations of 0% (control), 2%, 4% and 6%). The films were obtained by the casting method—the scheme of the process is presented in Figure 2. The film forming solution for the 2nd layer was made from 1% *w*/*v* furcellaran (FUR), to which, after dissolution (5 h, 100 °C), 1% *v*/*v* of glycerol was added. For the PA enhanced films, the PA extracts was added to the FUR solution to finally reach different concentrations (2%, 4%, 6%) in a volume of 250 mL. The control films did not contain the PA extract. The film forming solution was poured into the casting mold, and the layer was considered ready when the poured solution turned into a gel. At the same time, the film forming solution for the 1st layer was prepared by adding 2.5 g of gelatin to 250 mL of 1% *w*/*v* furcellaran with 1% *v*/*v* glycerol. The 1st layer film-forming solution was poured onto the 2nd layer. When the films became completely dry, they were removed from the molds, and further analyses were performed. Additionally, the content of the chosen compounds of the extract was evaluated (including betacyanin, betaxanthin and phenylpropanoids) [23].

### 3.2. Impact of Phytolacca americana Extract on Cell Cultures

#### 3.2.1. Cell Cultures

The PNT2 (ECACC 95012613) human prostate epithelial cells, Nthy-ori 3-1 (ECACC 90011609) thyroid follicular epithelial cells, hepatocellular carcinoma HepG2 (ATCC HB-8065) and HaCaT skin keratinocytes were used in the study. The cells were grown in standard conditions (37 °C, 5% CO_2_, relative humidity) and culture media (DMEM/F12 for PNT2, RPMI1640 for Nthy-ori 3-1, MEM for HepG2 and DMEM high glucose for HaCaT), supplemented with 10% FBS and antibiotics.

#### 3.2.2. Cell Viability Assay

Cell viability was determined after 24 and 48 h of incubation by MTT assay, as previously described [63]. The examined extract was dissolved in DMSO and then diluted in the culture medium to the working concentrations (from 0 to 500 μg/mL). The absorbance was measured at 570 nm using the Biotek Synergy microplate reader (BioTek Instruments Inc., Winooski, VT, USA). Three independent experiments were performed, and the results are expressed as cell viability as % of the control, untreated cells (mean ± SD) and IC_50_ values (concentration at which viability is inhibited by 50%).

### 3.3. Antimicrobial Properties of Films and Phytolacca americana Extract—In Vitro Analyses

#### 3.3.1. Films Assays

Three microorganisms (including two pathogens), *Staphylococcus aureus* ATCC 6538, *Escherichia coli* ATCC 8739 and *Saccharomyces cerevisiae*, were collected from the Department of Microbiology and Biomonitoring (UR Kraków, Kraków, Poland). For the assay, Petri dishes (⌀90 mm) were prepared with 10 mL of a solidified agar medium (Müller Hinton Agar 2 for bacteria and Sabouraud Glucose Agar for yeast (Biomaxima, Lublin, Poland)). The analyses were performed according to two different methodologies—one already described by Jamróz, et al. [29], referred to in this article as A1, and the second one, which included prolonged exposure of the films to water, denoted in this article as A2. For the A1 methodology, the1 cm × 1 cm film samples (previously sterilized under UV radiation for 30 min) were aseptically placed on the prepared agar medium. Then, a liquefied agar medium (~45 °C), inoculated with a standard suspension of microorganisms (optical density measured and adjusted to 0.5 MF (McFarland scale) using the DEN-1 McFarland Densitometer (BioSan, Riga, Latvia)), was poured and incubated at 37 °C for 24 h (the chosen temperature and time period for yeast was selected according to previous research [64]). To assess whether antimicrobial compounds are strongly bound in the film and show antimicrobial activity only after wetting and “loosening” the cross-linking, a second analysis was performed (A2). For this purpose, in sterile conditions, a piece of film (1 cm × 1 cm) was placed on a Petri dish (⌀90 mm) with a suitable medium (10 mL), and 0.5 mL of distilled water was sprinkled onto the film and left in room conditions for 24 h. After this time, the liquid medium with microbes was poured over it and placed in an incubator at 37 °C for 24 h. The analyses were carried out in triplicate. Visual analyses were performed to evaluate the growth of microorganisms in the areas around and above the films.

#### 3.3.2. Extract Assay

The disc diffusion method was performed for four different extract concentrations, 35 mg/mL (concentrated extract), 2.16 mg/mL, 1.44 mg/mL and 0.72 mg/mL, which were analogous concentrations to the films enriched with FUR + 2%PA/FUR + GEL, FUR + 4%PA/FUR + GEL and FUR + 6%PA/FUR + GEL, respectively. This analysis was conducted according to the recommendations of KORLD (National Reference Center for Microorganisms Susceptibility Testing) and EUCAST (The European Committee on Antimicrobial Susceptibility Testing). The extracts were pipetted onto aseptic discs in a volume of 20 µL. The analysis was carried out in triplicate.

### 3.4. Antioxidant Properties of Films

To determine the antioxidant potential of films, a new approach was assumed in order to maintain more natural conditions during the interaction of the films with oxidizing agents (e.g., 2,2-diphenyl-1picrylhydrazyl) and to determine whether the film itself, without prior exposure to external factors (humidity, increased temperature, etc.), would demonstrate antioxidant activity. The films were cut into small fragments (with an area not exceeding 10 mm^2^), which were used at further stages of analysis. Thus far, the antioxidant properties of films were estimated by previously preparing the liquid extract, which was then used for the next step of antioxidant analysis. The analyses were performed in three replications. The absorbance was measured at proper wavelength for each of the assays with Genesys 10 Vis Spectrophotometer (Thermo Scientific, Waltham, MA, USA).

#### 3.4.1. Ion Reduction Ability

Reduction power analysis of the films was performed according to the methods created by Benzie and Strain [65] (FRAP method) and by Apak et al. [44] (CUPRAC method). The analyses were conducted in accordance with already published methodology [23], with one modification—instead of a liquid film extract, 0.02 g of the solid film sample were added to the reagent (to maintain the representativity of the sample, each 0.02 g of sample were prepared from mixed small films fragments, made from a cut film sheet). The results of the assays were expressed as mmol of Trolox equivalents per 1 g of film (mM TExg^−1^) (Sigma-Aldrich, St. Louis, MO, USA).

#### 3.4.2. Free Radical Scavenging Activity

The radical scavenging capacity of the film was tested by the ability of neutralizing 2,2-diphenyl-1-picrylhydrazyl (DPPH) (Sigma-Aldrich, Poznań, Poland) and 2,2′-azino-bis(3-ethylbenzothiazoline-6-sulfonic acid (ABTS) (Sigma-Aldrich, Poznań, Poland) [66,67]. The analyses were performed according to already published methodology [23], with the same modification as previously specified. The results were expressed in percentages of DPPH^•^ or ABTS^2+•^ neutralization.

#### 3.4.3. Folin-Ciocâlteu Method

The reductive potential of films was determined according to already published methodology [23], with the same modification as previously specified. The final results were expressed as chlorogenic acid equivalents per 1 g of film (Sigma-Aldrich, Poznań Poland).

### 3.5. Determination Impact of the Film on African Catfish Quality During Storage

#### 3.5.1. Preparation of the African Catfish Samples

The evaluation of active packaging effectiveness was conducted on African catfish (*Clarias gariepinus*) samples. The catfish was produced from a local aquaculture farm (Gospodarstwo Rybackie Gady, Gady, Poland). The fish was purchased directly from the manufacturer in the form of fillets, and that form of fish was used as research material in this study. Approximately 5 kg catfish fillets (8 fish) were cut into small pieces with an approximate weight of 60 g. Then, the samples were randomly and equally divided into three groups, and each group contained 21 pieces of catfish fillets: control (C)—the synthetic film was used; experimental group 1 (A)—film with added pokeweed was applied (FUR + 6% PA/FUR + GEL); experimental group 2 (CA)—film without pokeweed was implemented (FUR/FUR + GEL). All samples were weighed. In addition, six samples were prepared for qualitative analyses at time 0. Then, the samples were packed in previously prepared C (synthetic film), A and CA films and stored in a climatic chamber (HPP 260eco, Memmert GmbH + Co. KG, Schwabach, Germany), protected from light, at 4 °C for 10 days. Measurements of physicochemical properties were carried out after the 2nd, 3rd, 4th, 5th, 6th and 10th days of storage. The microbiology was examined after 3, 6 and 10 days of storage.

#### 3.5.2. Determination of pH Value and Color Parameters

The pH of fish tissue was evaluated with an insertion electrode at three different points of each sample, using the portable TESTO 205 pH-meter (Testo, Titisee-Neustadt, Germany). Before measurements, the device was calibrated using 7.01 and 4.01 buffers.

Instrumental color was measured using the portable CR-400 chroma meter (Konica Minolta Sensing Inc., Osaka, Japan), at a 2° view angle, D65 illuminant, measurement/illumination area of φ 8 mm/φ 11 mm, calibrated prior to measurements with the use of a white tile standard. The color was evaluated directly on the fish samples in the CIE L*a*b* model [68]. Chroma (C*) and hue angle (*h*°) were calculated from equations:

$$C*=\sqrt{\left({a^{*}}^{2}+{b^{*}}^{2}\right)}$$

$$h{}^{\circ}=\tan^{-1}\frac{b^{*}}{a^{*}}\;\cdot \frac{180}{\Pi }$$

To determine changes in the fresh and stored fish samples, the coefficient ΔE (AMSA, 2012) was calculated according to the formula:

$$∆E=\sqrt{{\left({∆L}^{*}\right)}^{2}+{{(∆a}^{*})}^{2}+{{(∆b}^{*})}^{2}}$$

where ΔCIE L*, ΔCIE a* and ΔCIE b* denote the difference in the values of lightness, redness and yellowness, respectively, between fresh (0 day) and stored (2, 3, 4, 5, 6, 7, 10) fish samples. The difference in ΔE among the samples was classified as 0–0.5 (trace), 0.5–1.5 (slight), 1.5–3.0 (noticeable), 3.0–6.0 (appreciable), 6.0–12.0 (much), 12.0 or more (very much).

The redness index (*RI*) was calculated according to AMSA (2012) using Equation:

$$RI=\frac{a*}{b*}$$

#### 3.5.3. Sensory Evaluation

A panel consisting of eight judges, between the ages of 21 and 55, from the Department of Meat Technology and Chemistry, University of Warmia and Mazury in Olsztyn, were involved in the sensory evaluation. The panelists signed the Informed Consent forms for sensory evaluation, and ethical permission was not required, due to the fact that the panelists assessed the fish visually (they did not consume it). Sensory parameters (odor, color, surface and overall acceptability) of the samples were evaluated. After being unpacked from the film, the samples were presented in white polyethylene trays to each panelist, and a fresh catfish sample was offered to panelists for comparison purposes along with the test samples. The panelists gave scores for sensory characteristics, including color, odor, surface and overall acceptance, using a five-point descriptive scale, according to the method proposed by Ruan et al. [69], with some modifications. The color value expressed the degree of color darkening (towards gray, brown and purple) for the catfish samples: 5 = none; 4 = slight; 3 = small; 2 = moderate and 1 = extreme. The odor value represented the degree of spoilage odor regarding the catfish sample: 5 = none; 4 = slight; 3 = small; 2 = moderate and 1 = extreme. The surface value represented the degree of surface shine on the catfish samples: 5 = matte and 1 = intensely shiny. The overall acceptance value indicated the degree to which panelists liked the catfish sample as a whole, where 5 = like extremely and 1 = dislike extremely. Catfish was considered unacceptable when the score was below 3.

#### 3.5.4. Microbial Quality

In the examination of microbial growth over time, homogenized samples were made from 10 g of African catfish (*Clarias gariepinus*). Tissue was procured in a sterile manner. After weighing, it was then transferred to a 90 mL sterile saline solution (0.85% NaCl) and underwent homogenization for 60 s (Masticator Homogenizer Silver IUL S.A., Barcelona, Spain). Serial dilutions of the homogenate were undertaken, utilizing saline at a 1:10 (*v*/*v*) ratio. For the purpose of conducting microbial counts, a volume of 0.1 mL from each dilution was evenly spread onto the sterile media. *Enterobacterales* quantification was carried out using the VRBL agar (Merck, Darmstadt, Germany) for 24 h, at 37 °C, whereas the YGC agar (Merck, Darmstadt, Germany) was used for fungi assay. The samples were incubated at 22 °C for 72 h, and the LAB number was determined via the MRS agar (Merck, Darmstadt, Germany). These samples were incubated for 48 h at 30 °C in anaerobic conditions. Total viable cell (TVC) and psychrotroph counts were determined by dispensing 1.0 mL of the sample into a petri dish combined with 20 mL of a nutrient agar (Merck, Darmstadt, Germany) maintained at 50 °C. After solidification, the plates were incubated for 72 h at 30 °C for TVC and the psychrotrophs for 10 days at 6.5 °C. Microbial counts are expressed in colony-forming units per gram (CFU/g).

### 3.6. Statistical Analysis

In order to assess the significance of differences between the means, one-way analysis of variance and the Tukey NSD test were applied. The level of statistical significance was adopted as 0.05. Calculations were carried out using Statistica v. 12 software (Tibco, Palo Alto, Santa Clara, CA, USA). The significance of differences between the means regarding groups of catfish fillet physicochemical properties was assessed using Tukey’s reasonable significant difference (RIR) test, while variation components from advanced models were used between the means of discriminants in the sensory assessment, in which the fixed factor was the film used as well as the storage time, and the random factor was the panelist. The test results were presented as mean values ± standard error of the mean. In statistical analyses of microbial quality, the multiple *t*-test was used to compare the number of viable cells on the last day of storage, and two-way ANOVA was used to compare day-to-day differences.

## 4. Conclusions

The results obtained from the conducted antioxidant analysis indicate that films enriched with the PA extract are active films and could be used for food packaging to prolong the shelf life of products packed with them. Additionally, the results from antimicrobial in vitro analysis and in vivo African catfish storage assays indicate the possibility of using this type of packaging as an alternative to synthetic packaging films; however, further research needs to be done to determine the best method for extract formulation (including consideration of the active ingredient encapsulation method) and different polymer variances. Importantly, the results from cell culture analysis indicate that PA berry extracts are non-toxic for the tested human cell lines.

## Figures and Tables

**Table 1:** In vitro cytotoxic activity of methanol extract of fresh PA fruits after 24 h and 48 h of incubation against PNT2—prostate epithelial cells, HepG2—human liver cells, HaCaT—normal human keratinocytes and Nty-hori 3-1—normal human thyroid cells.

Incubation Time: 24 h				
Concentration [µg/mL]	Viable Cells ± SD [% of Control]			
	PNT2	HepG2	HaCaT	Nty-hori 3-1
25	89.2 ^a^ ± 3.1	89.0 ^a^ ± 1.2	95.0 ^a^ ± 3.3	98.5 ^a^ ± 5.8
50	92.1 ^a^ ± 2.4	83.6 ^a,b^ ± 2.1	89.7 ^a,b^ ± 1.9	92.7 ^a,b^ ± 5.6
100	91.2 ^a^ ± 2.8	84.0 ^a,b^ ± 3.7	88.2 ^a,b^ ± 2.6	90.7 ^a,b,c^ ± 1.6
200	87.6 ^a^ ± 4.3	82.9 ^a,b^ ± 4.2	82.9 ^b,c^ ± 2.7	85.3 ^b,c,d^ ± 3.2
300	88.7 ^a^ ± 2.9	80.0 ^a,b^ ± 5.1	81.9 ^b,c^ ± 3.2	80.9 ^c,d^ ± 1.2
400	69.9 ^b^ ± 5.2	75.4 ^b,c^ ± 2.0	77.8 ^c,d^ ± 3.0	79.4 ^c,d^ ± 2.7
500	66.0 ^b^ ± 4.6	69.6 ^c^ ± 2.6	72.7 ^d^ ± 3.8	81.2 ^d^ ± 4.6
IC_50_	>500	>500	>500	>500
**Incubation Time: 48 h**				
**Concentration [µg/mL]**	**Viable Cells ± SD [% of Control]**			
	**PNT2**	**HepG2**	**HaCaT**	**Nty-hori 3-1**
25	97.4 ^a^ ± 3.4	91.9 ^a,b^ ± 4.0	96.5 ^a^ ± 4.5	83.0 ^a^ ± 3.5
50	98.4 ^a^ ± 2.7	98.3 ^a^ ± 5.9	88.3 ^a,b^ ± 3.8	73.6 ^a,b^ ± 4.1
100	88.2 ^b^ ± 2.3	90.5 ^a,b^ ± 4.3	82.2 ^b,c^ ± 1.8	74.6 ^a,b^ ± 6.4
200	75.5 ^c^ ± 2.2	84.9 ^a,b^ ± 2.3	75.3 ^c,d^ ± 3.3	74.5 ^a,b^± 1.9
300	68.2 ^d^ ± 1.2	74.6 ^c^ ± 1.7	70.6 ^d,e^ ± 2.5	72.3 ^a,b,c^ ± 5.0
400	56.3 ^e^ ± 2.4	61.1 ^d^ ± 2.4	62.9 ^e,f^ ± 2.3	69.7 ^b,c^ ± 1.8
500	54.0 ^e^ ± 1.1	57.4 ^d^ ± 3.2	58.9 ^f^ ± 3.2	62.2 ^c^ ± 1.2
IC_50_	>500	>500	>500	>500

^a–f^—mean values in rows with different superscripts differ significantly at *p* < 0.05.

**Table 2:** Antimicrobial (performed according to A1 protocol) and antioxidant properties of films.

Antimicrobial Assay According to A1 Methodology				
		Film		
	FUR/FUR + GEL	FUR + 2% PA/FUR + GEL	FUR + 4% PA/FUR + GEL	FUR + 6% PA/FUR + GEL
Microorganism				
*S. aureus* ATCC 6538	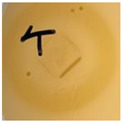	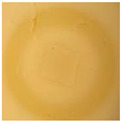	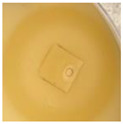	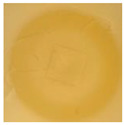
	Effect resembling “eagle effect”	Effect resembling “eagle effect”	Effect resembling “eagle effect”	Effect resembling “eagle effect”
*E. coli* ATCC 8739	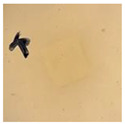	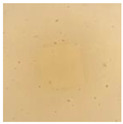	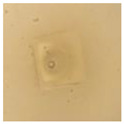	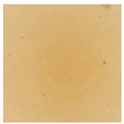
	No effect	No effect	No effect	No effect
*S. cerevisiae*	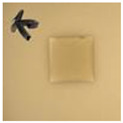	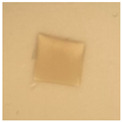	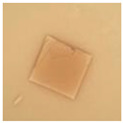	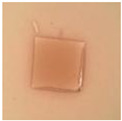
	No effect	No effect	No effect	No effect
**Antioxidant assays**				
**Method**				
FRAP [mM TE·g^−1^]	0.0 ^a^ ± 0.0	2.07 ^a,b^ ± 0.12	5.19 ^b^ ± 0.15	11.0 ^c^ ± 2.4
CUPRAC Cupric Reducing Antioxidant Power [mM TE·g^−1^]	20.0 ^a^ ± 1.5	8.3 ^c^ ± 1.1	3.8 ^b^ ± 0.9	4.5 ^b^ ± 1.9
DPPH radical scavenging activity [%]	5.4 ^a^ ± 0.7	7.9 ^a^ ± 1.7	7.0 ^a^ ± 2.3	5.9 ^a^ ± 2.0
ABTS radical scavenging activity [%]	30 ^a^ ± 4	61.2 ^b^ ± 2.8	72.8 ^c^ ± 2.4	71 ^c^ ± 5
Folin-Ciocâlteu [mg GAE/g]	5.02 ^a^ ± 0.05	21.7 ^b^ ± 1.8	29.5 ^c^ ± 2.8	35.8 ^d^ ± 1.7

Abbreviations: TE—Trolox equivalents, GAE—chlorogenic acid equivalents, letter K denotes control films; ^a–d^—mean values in rows with different superscripts differ significantly at *p* < 0.05.

**Table 3:** Effect of applied films and storage time on pH and color parameters of catfish fillets.

Attribute		Time (T, days)								*p* Value		
	Film (F)	0	1	2	3	4	5	6	10	F	T	F × T
pH	C	6.6 ± 0.2	6.34 ± 0.05	6.19 ± 0.03	6.56 ± 0.06	6.44 ± 0.05	6.33 ± 0.08	6.45 ± 0.1	6.13 ± 0.08	NS	*	NS
	CA	-	6.26 ± 0.11	6.49 ± 0.10	6.35 ± 0.07	6.29 ± 0.05	6.34 ± 0.07	6.52 ± 0.05	6.51 ± 0.09			
	A	-	6.35 ± 0.12	6.35 ± 0.04	6.49 ± 0.08	6.38 ± 0.16	6.40 ± 0.09	6.35 ± 0.07	6.53 ± 0.03			
L*	C	40.6 ± 1.3	40.6 ± 0.6	39.7 ± 0.5	40.4 ± 0.7	41.3 ± 1.2	42.9 ± 0.7	38.4 ± 0.9	39.7 ± 0.4	***	***	***
	CA	-	35.5 ± 0.3	37.2 ± 0.6	37.7 ± 0.5	36.2 ± 1.1	31.5 ± 0.8	32.4 ± 0.8	30.1 ± 0.4			
	A	-	27.13 ± 0.24	26.7 ± 0.4	27.1 ± 0.5	27.5 ± 0.8	27.2 ± 0.5	26.2 ± 0.8	26.5 ± 0.5			
a*	C	6.6 ± 0.3	4.1 ± 0.4	4.2 ± 0.4	4.4 ± 0.4	4.1 ± 0.6	5.4 ± 0.6	4.0 ± 0.6	7.2 ± 0.6	***	***	***
	CA	-	4.6 ± 0.2	5.0 ± 0.6	3.5 ± 0.4	3.8 ± 0.6	4.9 ± 0.4	5.0 ± 0.4	6.6 ± 0.4			
	A	-	18.7 ± 0.5	18.2 ± 0.9	15.4 ± 0.5	14.5 ± 0.6	11.1 ± 0.5	12.5 ± 0.6	10.32 ± 0.3			
b*	C	4.9 ± 0.3	2.93 ± 0.5	3.8 ± 0.3	4.9 ± 0.3	4.2 ± 0.6	4.7 ± 0.4	3.8 ± 0.5	4.5 ± 0.3	***	***	**
	CA	-	2.82 ± 0.14	3.1 ± 0.4	3.85 ± 0.20	4.1 ± 0.6	2.85 ± 0.18	3.18 ± 0.16	3.71 ± 0.17			
	A	-	1.55 ± 0.13	−1.7 ± 0.20	−1.53 ± 0.1	0.79 ± 0.10	1.17 ± 0.19	1.27 ± 0.2	1.59 ± 0.16			
C*	C	7.8 ± 0.4	5.1 ± 0.6	5.8 ± 0.5	6.6 ± 0.5	6.0 ± 0.7	7.2 ± 0.7	5.7 ± 0.5	4.5 ± 0.3	***	***	***
	CA	-	5.41 ± 0.27	5.9 ± 0.6	5.2 ± 0.4	5.8 ± 0.6	5.7 ± 0.4	6.0 ± 0.4	7.6 ± 0.6			
	A	-	18.8 ± 0.5	18.3 ± 0.9	15.5 ± 0.5	14.5 ± 0.6	11.2 ± 0.6	12.6 ± 0.6	10.4 ± 0.26			
h°	C	33.0 ± 1.0	34 ± 3	42.0 ± 2.7	48.6 ± 2.0	47 ± 5	41.5 ± 2.6	44 ± 5	32.1 ± 1.8	***	***	***
	CA	-	31.6 ± 1.0	32 ± 3	49.4 ± 2.7	50 ± 6	30.4 ± 1.1	32.8 ± 1.5	30.6 ± 2.7			
	A	-	4.8 ± 0.5	354.3 ± 1.0	354.3 ± 0.5	3.2 ± 0.5	5.9 ± 0.8	5.9 ± 1.1	8.7 ± 0.8			
|RI|	C	1.55 ± 0.1	1.42 ± 0.10	1.13 ± 0.14	0.90 ± 0.08	0.97 ± 0.09	1.14 ± 0.15	1.04 ± 0.15	1.63 ± 0.16	***	***	***
	CA	-	1.64 ± 0.07	1.59 ± 0.08	0.90 ± 0.12	0.93 ± 0.09	1.72 ± 0.06	1.59 ± 0.14	1.78 ± 0.25			
	A	-	12.2 ± 1.0	10.8 ± 0.6	10.3 ± 0.9	19 ± 3	9.5 ± 0.6	10.0 ± 0.8	6.6 ± 0.6			
ΔE	C	-	3.4 ± 0.5	3.1 ± 0.4	3.14 ± 0.27	3.5 ± 0.06	3.3 ± 1.7	3.7 ± 0.6	3.1 ± 1.1	***	*	**
			appreciable									
	CA	-	5.7 ± 1.2	4.2 ± 1.2	4.6 ± 1.1	5.5 ± 1.6	9.4 ± 0.8	8.5 ± 0.4	10.6 ± 1.6			
			appreciable				much					
	A	-	18.4 ± 1.0	19.2 ± 0.8	17.3 ± 0.9	15.8 ± 0.9	14.6 ± 1.0	15.9 ± 1.2	15.7 ± 1.8			
			very much									

*** significant difference at *p* < 0.001. ** significant difference at *p* < 0.01. * significant difference at *p* < 0.05. NS—non-significant difference at *p* > 0.05. C—control; CA—film without pokeweed; A—film with added pokeweed.

**Table 4:** Sensory evaluation.

Attribute			Time (T, days)							*p* Value		
		Film (F)	0	1	2	3	4	5	6	F	T	F × T
**Color scores**	dark and gray	C	5.0 ± 0.0	4.17 ± 0.09	3.67 ± 0.18	3.71 ± 0.14	3.72 ± 0.14	3.95 ± 0.19	3.62 ± 0.16	NS	*	NS
		CA	-	5.0 ± 0.0	4.00 ± 0.20	3.19 ± 0.19	3.86 ± 0.21	3.33 ± 0.14	2.86 ± 0.19			
		A	-	5.0 ± 0.0	5.0 ± 0.0	4.00 ± 0.24	3.72 ± 0.23	4.29 ± 0.12	2.9 ± 0.14			
	dark and brown	C	5.0 ± 0.0	5.0 ± 0.0	3.67 ± 0.11	4.71 ± 0.10	3.78 ± 0.15	3.91 ± 0.10	3.00 ± 0.10	***	***	***
		CA	-	4.11 ± 0.08	3.67 ± 0.13	3.67 ± 0.13	3.38 ± 0.18	3.14 ± 0.13	2.86 ± 0.19			
		A	-	5.0 ± 0.0	4.22 ± 0.31	3.00 ± 0.18	3.44 ± 0.25	4.29 ± 0.12	2.43 ± 0.16			
	purple	C	5.0 ± 0.0	4.3 ± 0.4	5.0 ± 0.0	4.95 ± 0.05	4.83 ± 0.09	4.81 ± 0.19	4.95 ± 0.05	***	***	***
		CA	-	5.0 ± 0.0	5.0 ± 0.0	5.0 ± 0.0	5.0 ± 0.0	4.71 ± 0.10	5.0 ± 0.0			
		A	-	1.0 ± 0.0	1.0 ± 0.0	1.05 ± 0.05	1.9 ± 0.4	1.48 ± 0.16	1.19 ± 0.15			
**Odor scores**		C	5.0 ± 0.0	5.0 ± 0.0	5.0 ± 0.0	4.71 ± 0.10	4.28 ± 0.16	2.6 ± 0.3	1.38 ± 0.13	***	***	**
		CA	-	5.0 ± 0.0	4.68 ± 0.11	4.62 ± 0.15	4.29 ± 0.12	3.00 ± 0.24	2.10 ± 0.15			
		A	-	5.0 ± 0.0	5.0 ± 0.0	4.71 ± 0.10	4.50 ± 0.12	2.86 ± 0.16	3.1 ± 0.21			
**Surface**		C	4.22 ± 0.10	4.06 ± 0.06	4.0 ± 0.0	4.14 ± 0.19	4.11 ± 0.18	4.0 ± 0.15	3.91 ± 0.12	***	***	***
		CA	-	4.94 ± 0.06	4.33 ± 0.11	4.33 ± 0.19	4.29 ± 0.17	4.62 ± 0.11	3.95 ± 0.16			
		A	-	4.67 ± 0.11	4.94 ± 0.06	3.95 ± 0.23	4.50 ± 0.15	4.33 ± 0.13	4.43 ± 0.16			
**Overall acceptance**		C	5.0 ± 0.0	5.0 ± 0.0	4.22 ± 0.13	4.10 ± 0.10	3.39 ± 0.28	2.24 ± 0.19	1.38 ± 0.11	***	***	***
		CA	-	4.17 ± 0.09	4.0 ± 0.0	3.76 ± 0.14	3.14 ± 0.20	2.33 ± 0.19	2.00 ± 0.12			
		A	-	4.67 ± 0.11	3.67 ± 0.11	3.57 ± 0.20	3.00 ± 0.23	2.24 ± 0.17	2.57 ± 0.20			

*** significant difference at *p* < 0.001. ** significant difference at *p* < 0.01. * significant difference at *p* < 0.05. NS—non-significant difference at *p* > 0.05. C—control; CA—film without pokeweed; A—film with added pokeweed.

## Data Availability

The raw data supporting the conclusions of this article will be made available by the authors on request.
